# 4,6,7,9,10,12-Hexahydro-1,3-dithiolo[4,5-*f*][1,4,9]oxadithia­cyclo­undecine-2-thione

**DOI:** 10.1107/S1600536809024003

**Published:** 2009-06-27

**Authors:** Rui-Bin Hou, Bao Li, Bing-Zhu Yin, Li-Xin Wu

**Affiliations:** a Key Laboratory of Organism Functional Factors of Changbai Mountain, Yanbian University, Ministry of Education, Yanji 133002, People’s Republic of China; b State Key Laboratory of Supramolecular Structure and Materials, College of Chemistry, Jilin University, Changchun 130012, People’s Republic of China

## Abstract

In the title mol­ecule, C_9_H_12_S_5_O, the five-membered ring and attached S atom are essentially coplanar [mean deviation from the mean plane = 0.020 (1) Å]. The two S atoms belonging to the macrocycle deviate from this plane by 1.005 (1) and 1.337 (2) Å. In the crystal, π–π inter­actions link the mol­ecules into centrosymmetric dimers with a short distance of 3.753 (5) Å between the centroids of the five-membered rings.

## Related literature

The title compound was prepared according to Chen *et al.* (2005 ▶). For background literature concerning crown-ether-annulated 1,3-dithiol-2-thione derivatives, see: Hansen *et al.* (1992 ▶); Trippé *et al.* (2002 ▶).
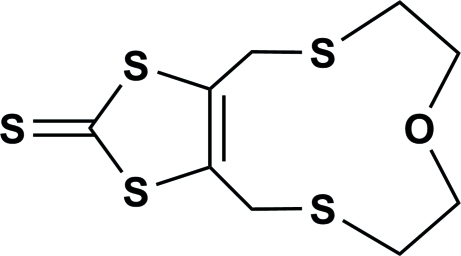


## Experimental

### 

#### Crystal data

C_9_H_12_OS_5_
*M* *_r_* = 296.49
Triclinic, 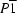

*a* = 8.3425 (17) Å
*b* = 8.9611 (18) Å
*c* = 9.820 (2) Å
α = 98.10 (3)°
β = 106.58 (3)°
γ = 112.74 (3)°
*V* = 622.2 (2) Å^3^
*Z* = 2
Mo *K*α radiation
μ = 0.90 mm^−1^
*T* = 291 K
0.15 × 0.12 × 0.12 mm

#### Data collection

Rigaku R-AXIS RAPID diffractometer
Absorption correction: multi-scan (*ABSCOR*; Higashi, 1995 ▶) *T*_min_ = 0.877, *T*_max_ = 0.900
6141 measured reflections
2813 independent reflections
2560 reflections with *I* > 2σ(*I*)
*R*_int_ = 0.017

#### Refinement

*R*[*F* ^2^ > 2σ(*F* ^2^)] = 0.031
*wR*(*F* ^2^) = 0.106
*S* = 1.06
2813 reflections
136 parameters
H-atom parameters constrained
Δρ_max_ = 0.41 e Å^−3^
Δρ_min_ = −0.36 e Å^−3^

### 

Data collection: *RAPID-AUTO* (Rigaku, 1998 ▶); cell refinement: *RAPID-AUTO*; data reduction: *CrystalStructure* (Rigaku/MSC, 2002 ▶); program(s) used to solve structure: *SHELXS97* (Sheldrick, 2008 ▶); program(s) used to refine structure: *SHELXL97* (Sheldrick, 2008 ▶); molecular graphics: *PLATON* (Spek, 2009 ▶); software used to prepare material for publication: *SHELXL97*.

## Supplementary Material

Crystal structure: contains datablocks global, I. DOI: 10.1107/S1600536809024003/cv2573sup1.cif

Structure factors: contains datablocks I. DOI: 10.1107/S1600536809024003/cv2573Isup2.hkl

Additional supplementary materials:  crystallographic information; 3D view; checkCIF report

## Figures and Tables

**Fig. 1.**
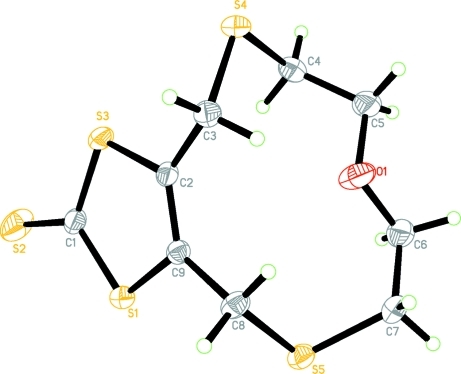
The molecular structure of (I) showing the atom numbering. Displacement ellipsoids of non-H atoms are drawn at the 30% probalility level.

## supplementary crystallographic information

### Comment

Crown ether annulated 1,3-dithiol-2-thione derivatives have been intensively investigated as key intermediate of the crowned tetrathiafulvalenes because the latter molecules show electrochemical signaling for various metal cations (Hansen *et al.*,1992; Trippé *et al.*, 2002). We report hererin the crystal structure of the title compound, (I).

In (I) (Fig. 1),  five-membered ring and attached S2 atom are essentially coplanar with the mean deviation of 0.020 (1) Å from the mean plane  *P*. The plane defined by the rest non-hydrogen atoms forms an angle of 70.25 (4) ° with  *P*. The π-π interactions with the short distance of 3.753 (5) Å between the centroids of five-membered rings link the molecules into centrosymmetric dimers.

### Experimental

The title compound was prepared according to the literature (Chen *et al.*, 2005). Single crystals suitable for X-ray diffraction were prepared by slow evaporation a mixture of dichloromethane and petroleum at room temperatue.

### Refinement

Carbon-bound H-atoms were placed in calculated positions with C—H 0.97 Å and were included in the refinement in the riding model, with *U*_iso_(H) = 1.2 *U*_eq_(C).

### Crystal data

**Table:**

C_9_H_12_OS_5_	*Z* = 2
*M**_r_* = 296.49	*F*(000) = 308
Triclinic, *P*1	*D*_x_ = 1.583 Mg m^−^^3^
Hall symbol: -P 1	Mo *K*α radiation, λ = 0.71073 Å
*a* = 8.3425 (17) Å	Cell parameters from 5678 reflections
*b* = 8.9611 (18) Å	θ = 3.4–27.0°
*c* = 9.820 (2) Å	µ = 0.90 mm^−^^1^
α = 98.10 (3)°	*T* = 291 K
β = 106.58 (3)°	Block, yellow
γ = 112.74 (3)°	0.15 × 0.12 × 0.12 mm
*V* = 622.2 (2) Å^3^	

### Data collection

**Table:**

Rigaku R-AXIS RAPID diffractometer	2813 independent reflections
Radiation source: fine-focus sealed tube	2560 reflections with *I* > 2σ(*I*)
graphite	*R*_int_ = 0.017
ω scans	θ_max_ = 27.5°, θ_min_ = 3.4°
Absorption correction: multi-scan (*ABSCOR*; Higashi, 1995)	*h* = −10→10
*T*_min_ = 0.877, *T*_max_ = 0.900	*k* = −11→11
6141 measured reflections	*l* = −12→12

### Refinement

**Table:**

Refinement on *F*^2^	Primary atom site location: structure-invariant direct methods
Least-squares matrix: full	Secondary atom site location: difference Fourier map
*R*[*F*^2^ > 2σ(*F*^2^)] = 0.031	Hydrogen site location: inferred from neighbouring sites
*wR*(*F*^2^) = 0.106	H-atom parameters constrained
*S* = 1.06	*w* = 1/[σ^2^(*F*_o_^2^) + (0.0645*P*)^2^ + 0.2783*P*] where *P* = (*F*_o_^2^ + 2*F*_c_^2^)/3
2813 reflections	(Δ/σ)_max_ = 0.003
136 parameters	Δρ_max_ = 0.41 e Å^−^^3^
0 restraints	Δρ_min_ = −0.36 e Å^−^^3^

### Special details

**Table:**

Experimental. (See detailed section in the paper)
Geometry. All e.s.d.'s (except the e.s.d. in the dihedral angle between two l.s. planes) are estimated using the full covariance matrix. The cell e.s.d.'s are taken into account individually in the estimation of e.s.d.'s in distances, angles and torsion angles; correlations between e.s.d.'s in cell parameters are only used when they are defined by crystal symmetry. An approximate (isotropic) treatment of cell e.s.d.'s is used for estimating e.s.d.'s involving l.s. planes.
Refinement. Refinement of *F*^2^ against ALL reflections. The weighted *R*-factor *wR* and goodness of fit *S* are based on *F*^2^, conventional *R*-factors *R* are based on *F*, with *F* set to zero for negative *F*^2^. The threshold expression of *F*^2^ > σ(*F*^2^) is used only for calculating *R*-factors(gt) *etc*. and is not relevant to the choice of reflections for refinement. *R*-factors based on *F*^2^ are statistically about twice as large as those based on *F*, and *R*- factors based on ALL data will be even larger.

### Fractional atomic coordinates and isotropic or equivalent isotropic displacement parameters (Å^2^)

**Table:**

	*x*	*y*	*z*	*U*_iso_*/*U*_eq_	
C1	0.3295 (3)	0.6916 (3)	0.4927 (2)	0.0376 (4)	
C2	0.2130 (3)	0.8764 (2)	0.3463 (2)	0.0300 (4)	
C3	0.1113 (3)	0.9792 (2)	0.3073 (2)	0.0354 (4)	
H3A	0.1194	1.0051	0.2161	0.042*	
H3B	0.1724	1.0851	0.3848	0.042*	
C4	−0.2203 (3)	0.6799 (3)	0.1413 (3)	0.0408 (5)	
H4A	−0.1445	0.6233	0.1746	0.049*	
H4B	−0.3469	0.6075	0.1315	0.049*	
C5	−0.2231 (3)	0.6956 (3)	−0.0092 (3)	0.0486 (5)	
H5A	−0.2975	0.5852	−0.0811	0.058*	
H5B	−0.2805	0.7677	−0.0378	0.058*	
C6	−0.0193 (3)	0.6766 (3)	−0.1285 (3)	0.0466 (5)	
H6A	−0.1108	0.6664	−0.2208	0.056*	
H6B	−0.0423	0.5639	−0.1211	0.056*	
C7	0.1742 (3)	0.7686 (3)	−0.1276 (2)	0.0439 (5)	
H7A	0.1991	0.8846	−0.1229	0.053*	
H7B	0.1754	0.7190	−0.2215	0.053*	
C8	0.4004 (3)	0.9279 (2)	0.1782 (2)	0.0367 (4)	
H8A	0.5328	1.0056	0.2243	0.044*	
H8B	0.3342	0.9920	0.1443	0.044*	
C9	0.3363 (2)	0.8552 (2)	0.2922 (2)	0.0294 (4)	
O1	−0.0377 (2)	0.7650 (2)	−0.0088 (2)	0.0600 (5)	
S1	0.43959 (7)	0.73453 (6)	0.36855 (5)	0.03533 (15)	
S2	0.36763 (14)	0.58331 (12)	0.60929 (8)	0.0708 (3)	
S3	0.17163 (8)	0.77394 (7)	0.48048 (6)	0.03867 (15)	
S4	−0.13328 (7)	0.87314 (7)	0.28369 (6)	0.04162 (16)	
S5	0.36387 (8)	0.77082 (7)	0.01819 (6)	0.04019 (15)	

### Atomic displacement parameters (Å^2^)

**Table:**

	*U*^11^	*U*^22^	*U*^33^	*U*^12^	*U*^13^	*U*^23^
C1	0.0448 (11)	0.0454 (11)	0.0277 (9)	0.0247 (9)	0.0138 (8)	0.0110 (8)
C2	0.0293 (8)	0.0333 (9)	0.0239 (8)	0.0121 (7)	0.0089 (7)	0.0061 (7)
C3	0.0375 (10)	0.0361 (9)	0.0347 (10)	0.0190 (8)	0.0136 (8)	0.0087 (8)
C4	0.0313 (9)	0.0420 (10)	0.0486 (12)	0.0163 (8)	0.0140 (9)	0.0140 (9)
C5	0.0318 (10)	0.0615 (14)	0.0431 (12)	0.0164 (10)	0.0121 (9)	0.0057 (10)
C6	0.0439 (11)	0.0526 (12)	0.0361 (11)	0.0199 (10)	0.0127 (9)	0.0023 (9)
C7	0.0506 (12)	0.0565 (13)	0.0329 (10)	0.0266 (10)	0.0214 (9)	0.0172 (9)
C8	0.0388 (10)	0.0339 (9)	0.0361 (10)	0.0107 (8)	0.0200 (8)	0.0097 (8)
C9	0.0283 (8)	0.0289 (8)	0.0276 (9)	0.0101 (7)	0.0105 (7)	0.0059 (7)
O1	0.0356 (8)	0.0692 (11)	0.0481 (10)	0.0048 (8)	0.0198 (7)	−0.0146 (8)
S1	0.0340 (3)	0.0422 (3)	0.0348 (3)	0.0204 (2)	0.0152 (2)	0.0112 (2)
S2	0.1147 (7)	0.0990 (6)	0.0578 (4)	0.0820 (6)	0.0517 (4)	0.0521 (4)
S3	0.0443 (3)	0.0543 (3)	0.0322 (3)	0.0285 (2)	0.0224 (2)	0.0188 (2)
S4	0.0399 (3)	0.0544 (3)	0.0438 (3)	0.0303 (2)	0.0214 (2)	0.0135 (2)
S5	0.0468 (3)	0.0511 (3)	0.0357 (3)	0.0300 (2)	0.0217 (2)	0.0135 (2)

### Geometric parameters (Å, °)

**Table:**

C1—S2	1.642 (2)	C5—H5A	0.9700
C1—S1	1.726 (2)	C5—H5B	0.9700
C1—S3	1.726 (2)	C6—O1	1.402 (3)
C2—C9	1.346 (3)	C6—C7	1.499 (3)
C2—C3	1.495 (3)	C6—H6A	0.9700
C2—S3	1.7471 (19)	C6—H6B	0.9700
C3—S4	1.814 (2)	C7—S5	1.796 (2)
C3—H3A	0.9700	C7—H7A	0.9700
C3—H3B	0.9700	C7—H7B	0.9700
C4—C5	1.498 (3)	C8—C9	1.497 (3)
C4—S4	1.802 (2)	C8—S5	1.820 (2)
C4—H4A	0.9700	C8—H8A	0.9700
C4—H4B	0.9700	C8—H8B	0.9700
C5—O1	1.426 (3)	C9—S1	1.747 (2)
			
S2—C1—S1	124.30 (13)	O1—C6—H6A	109.7
S2—C1—S3	123.21 (13)	C7—C6—H6A	109.7
S1—C1—S3	112.49 (12)	O1—C6—H6B	109.7
C9—C2—C3	127.67 (18)	C7—C6—H6B	109.7
C9—C2—S3	115.53 (15)	H6A—C6—H6B	108.2
C3—C2—S3	116.79 (14)	C6—C7—S5	117.34 (17)
C2—C3—S4	112.96 (14)	C6—C7—H7A	108.0
C2—C3—H3A	109.0	S5—C7—H7A	108.0
S4—C3—H3A	109.0	C6—C7—H7B	108.0
C2—C3—H3B	109.0	S5—C7—H7B	108.0
S4—C3—H3B	109.0	H7A—C7—H7B	107.2
H3A—C3—H3B	107.8	C9—C8—S5	114.07 (14)
C5—C4—S4	116.74 (17)	C9—C8—H8A	108.7
C5—C4—H4A	108.1	S5—C8—H8A	108.7
S4—C4—H4A	108.1	C9—C8—H8B	108.7
C5—C4—H4B	108.1	S5—C8—H8B	108.7
S4—C4—H4B	108.1	H8A—C8—H8B	107.6
H4A—C4—H4B	107.3	C2—C9—C8	127.63 (18)
O1—C5—C4	110.55 (19)	C2—C9—S1	116.14 (15)
O1—C5—H5A	109.5	C8—C9—S1	116.20 (14)
C4—C5—H5A	109.5	C6—O1—C5	113.57 (18)
O1—C5—H5B	109.5	C1—S1—C9	97.73 (10)
C4—C5—H5B	109.5	C1—S3—C2	98.01 (10)
H5A—C5—H5B	108.1	C4—S4—C3	102.59 (10)
O1—C6—C7	109.68 (19)	C7—S5—C8	103.70 (11)
